# The effect of isometric exercise training on arterial stiffness: A randomized crossover controlled study

**DOI:** 10.14814/phy2.15690

**Published:** 2023-05-19

**Authors:** Jamie J. Edwards, Navazh Jalaludeen, Arian Beqiri, Jonathan D. Wiles, Rajan Sharma, Jamie M. O'Driscoll

**Affiliations:** ^1^ School of Psychology and Life Sciences Canterbury Christ Church University Canterbury UK; ^2^ Cambridge Clinical Trials Unit Cambridge University Hospitals NHS Foundation Trust Cambridge UK; ^3^ Faculty of Life Sciences and Medicine Kings College London, School of Biomedical Engineering and Imaging Services London UK; ^4^ Department of Cardiology St George's University Hospitals NHS Foundation Trust London UK

**Keywords:** augmentation index, blood pressure, hypertension, isometric exercise training

## Abstract

Isometric exercise training (IET) is an effective intervention for the management of resting blood pressure (BP). However, the effects of IET on arterial stiffness remain largely unknown. Eighteen unmedicated physically inactive participants were recruited. Participants were randomly allocated in a cross‐over design to 4 weeks of home‐based wall squat IET and control period, separated by a 3‐week washout period. Continuous beat‐to‐beat hemodynamics, including early and late systolic (sBP 1 and sBP 2, respectively) and diastolic blood pressure (dBP) were recorded for a period of 5 min and waveforms were extracted and analyzed to acquire the augmentation index (AIx) as a measure of arterial stiffness. sBP 1 (−7.7 ± 12.8 mmHg, *p* = 0.024), sBP 2 (−5.9 ± 9.9 mmHg, *p* = 0.042) and dBP (−4.4 ± 7.2 mmHg, *p* = 0.037) all significantly decreased following IET compared to the control period. Importantly, there was a significant reduction in AIx following IET (−6.6 ± 14.5%, *p* = 0.02) compared to the control period. There were also adjacent significant reductions in total peripheral resistance (−140.7 ± 65.8 dynes·cm‐5, *p* = 0.042) and pulse pressure (−3.8 ± 4.2, *p* = 0.003) compared to the control period. This study demonstrates an improvement in arterial stiffness following a short‐term IET intervention. These findings have important clinical implications regarding cardiovascular risk. Mechanistically, these results suggest that reductions in resting BP following IET are induced via favorable vascular adaptations, although the intricate details of such adaptations are not yet clear.

## 1 INTRODUCTION

As a primary risk factor for hypertension, arterial stiffness is an independent marker of cardiovascular disease (Boutouyrie et al., 2021). Given its close association with the progression of atherosclerosis (Van Popele et al., 2001), risk of stroke and ischaemic heart disease (Mattace‐Raso et al., 2006), and importantly, its prognostic implications for all‐cause mortality (Vlachopoulos, Aznaouridis & Stefanadis, 2010; Vlachopoulos, Aznaouridis, O'Rourke, et al., 2010), arterial stiffness remains a primary target for therapeutic intervention.

Isometric exercise training (IET) is a highly effective and time‐efficient anti‐hypertensive intervention, with multiple meta‐analyses demonstrating statistically and clinically significant reductions in resting blood pressure (BP) (Edwards et al., 2022; Inder et al., 2016; López‐Valenciano et al., 2019). Fundamentally, these BP reductions have been largely attributed to changes in peripheral vascular resistance, rather than adjustments to central cardiac output (de Caux et al., 2021; Taylor et al., 2019). Despite this, the effects of IET on parameters of arterial stiffness remain largely unknown. Indeed, only a few trials have investigated vascular adaptations following IET, reporting mixed results (Cahu Rodrigues et al., 2020; Correia et al., 2020).

The augmentation index (AIx), which is derived from pulse‐wave reflection, provides an independent index of systemic arterial stiffness (Fantin et al., 2007). Indeed, previous work has reported that the AIx is closely associated with cardiovascular risk (Janner et al., 2012; Nürnberger et al., 2002) and all‐cause mortality (Janner et al., 2013). As such, calculation of the AIx following a programme of IET may further support the clinical utility of IET as a preventative intervention, as well as provide important mechanistic insight into the BP reductions seen following IET. Previous research has demonstrated the validity of peripherally acquired AIx (Munir et al., 2008). Therefore, the aims of this study were to investigate changes in the early and late systolic BP (sBP 1 and sBP 2, respectively), diastolic BP (dBP) and peripheral AIx following a 4 weeks of home‐based wall squat IET intervention.

## 2 METHODOLOGY

### 2.1 Study population and ethical approval

Eighteen males (mean age 45 ± 5.9; height 176.5 ± 5.8 cm; weight 86.6 ± 11.5 kg) who self‐reported as physically inactive, volunteered to participate in this prospective, single centre randomized cross‐over controlled trial. Participants were normotensive in accordance with the current European guidelines (Williams et al., 2018) and were not under any acute or chronic pharmacotherapy, including antibiotics. All participants presented with a normal clinical cardiovascular examination and 12‐lead electrocardiogram (ECG) with no cardiac or metabolic disease. None of the participants were current smokers. This research study conformed to the Declaration of Helsinki principles and was approved by the local ethics committee (Ref:12/SAS/122). Written informed consent was obtained from all participants before testing. This work is a sub‐study of research previously published (Taylor et al., 2019), which includes a flow chart for participant recruitment.

### 2.2 Study procedures

All eligible participants were recruited and randomized in a crossover design to a 4‐week IET intervention or control period separated by a 3‐week washout period. Previous de‐training data demonstrated that the significant BP reductions following IET were mitigated within 10 days following the final session, which suggests 3 weeks will constitute a sufficient washout period (Howden et al., 2002). Block randomization was performed via Microsoft Excel. All participants were required to attend the Canterbury Christ Church University laboratory on five separate occasions. The first visit for the IET component was required in order to prescribe an individualized IET intensity wall squat training angle, using the incremental isometric wall squat test, as previously described (Taylor et al., 2019). The remaining sessions were dedicated to the acquisition of the relevant cardiovascular parameters before and after the intervention and control period. Post‐intervention data collection occurred at the same time of the day as the pre‐intervention testing and within 3 days of the final IET session. Participants were required to maintain normal circadian and dietary patterns throughout the study and were also asked to refrain from alcohol and caffeine consumption for 24 h as well as fast for at least 4 h before laboratory testing. None of the lab investigators or participants were blinded to group assignment; however, AIx analysis was performed via an independent blinded investigator.

### 2.3 Resting clinic blood pressure

Resting clinic brachial artery BP was recorded in a temperature‐controlled laboratory according to current guidelines (Williams et al., 2018). Measures were taken before and after the IET intervention and control period using a validated automated device (Dinamap Pro 200 Critikon; GE Medical Systems).

### 2.4 Continuous blood pressure and augmentation index

Hemodynamic data including continuous sBP and dBP, resting stroke volume (SV), resting heart rate (HR), pulse pressure (PP), and total peripheral resistance (TPR) was acquired using the Task Force® Monitor (TFM), which is a validated non‐invasive beat‐to‐beat monitoring system. Hemodynamic measures were acquired over a 5‐min period via impedance cardiography, with continuous beat‐to‐beat BP acquired via the vascular unloading technique at the proximal limb of the index or middle finger. These data was automatically corrected to oscilliometric BP values obtained at the brachial artery of the opposite arm. Raw data of the entire 5‐min continuous BP recording was analyzed for each participant using a bespoke analysis pipeline created in MATLAB R2021a. The pipeline was used to find the sBP and dBP troughs for each heartbeat, and discard any data that was too noisy to analyze, which comprised the following steps:

1. Using the MATLAB ‘findpeaks’ function from the signal processing toolbox, search for peaks with a sharp prominence. Take the mean distance between these and then rerun the search for peaks ensuring a minimum distance between peaks of 0.33 of the mean distance. This minimizes the chance of being distorted by sBP peaks that are too close to one another.
2. Take the negative of the BP raw signal and run the ‘findpeaks’ function on these to find the dBP troughs.
3. Loop through the indices for the sBP peaks and take each cycle to be defined as the recorded BP points between sBP peaks. Check that the second peak (which should be the second sBP1) is not significantly larger than the second peak—if it is, then skip to the next index.
4. Check if there is a single peak between these, including the second point, which will correspond to sBP2.
5. If a clear prominence is not found, then looking at the second derivatives of the raw BP signal in the heartbeat, find the index where this is at its minimum. This will provide the sBP2 peak.

**FIGURE 1:**
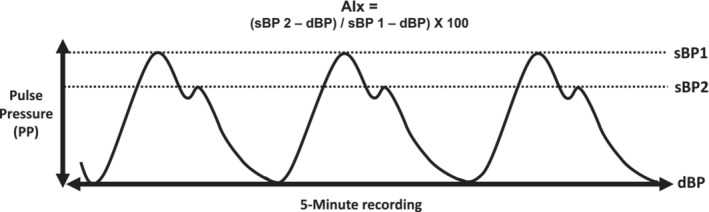
Example tracing of blood pressure waveforms and subsequent calculation of AIx.

The AIx was then calculated as follows: (late peak sBP [sBP 2]—dBP)/(early peak of sBP [sBP 1]—dBP) × 100 (%) (Kohara et al., 2005) (Figure 1). Heart rate was recorded through a 6‐channel ECG and a 5‐min average was used for analysis.

HR was acquired through a six‐channel ECG and SV was recorded via impedance cardiography through three electrode bands, two of which were adjacent to the thorax, in line with the xiphoid process, while the other was positioned on the nape of the neck. TPR was calculated in accordance with Ohm's law, while CO was automatically derived from HR and SV. Both TPR and CO were indexed to body surface area via the Du Bois method (Du Bois & Du Bois, 1916).

### 2.5 Isometric exercise training intervention

The IET intervention consisted of a 4‐week unsupervised home‐based wall squat programme, comprising of 4 × 2 min bouts, separated by 2 min rest intervals, performed three times per week (12 IET sessions in total). Individualized intensity prescription was based on the participant's knee joint angle acquired from the incremental test to elicit 95% peak HR. All participants recorded HR data following each IET bout and uploaded their full session data to a personal online database which was monitored by the investigators. The decision to prescribe IET based on HR was influenced by the safety and practicality limitations of performing 1‐repetition maximum/maximal voluntary contraction squat tests in untrained participants. As such, the close monitoring of HR allowed for practical and effective intensity monitoring throughout the intervention based on the responses observed in the initial incremental test. Adjacent to HR, participants also recorded a rate of perceived exertion score for each wall squat, which was concurrently utilized in the optimization of IET intensity. During the control period, participants were required to maintain their normal routine and daily activities.

## 3 SAMPLE SIZE CALCULATION

As a sub‐study of Taylor et al. (2019) the sample size of this work was calculated to identify statistically and clinically significant BP changes. Based on previous research in which a home‐based IET programme was performed, we expect a minimum BP reduction of 5 mmHg in the IET group with no statistically significant change in the control group (Wiles et al., 2010). Given the likely change (~4.3%) and the coefficient of variation of sBP (4.6%) from Wiles et al. (2010) we estimated a sample size of 18 participants with 80% power and a *p*‐value < 0.05.

### 3.1 Statistical analysis

Continuous variables are expressed as mean ± SD. Analysis of Covariance was performed on change scores (postpre) for the two conditions, with the order of the intervention included as a covariate in the analysis. AIx was adjusted for mean BP. All data were analyzed using the statistical package for social sciences (SPSS 26 release version for Windows; SPSS Inc., Chicago, Illinois, USA).

## 4 RESULTS

There were no study withdrawals and participants reported 100% adherence to the intervention. There were no adverse events during or following any IET session. At baseline, there were no significant differences between the groups for any participant characteristics. One of the study participants' data was corrupted upon transfer; therefore, AIx analysis was only performed on 17‐participants.

### 4.1 Hemodynamics and augmentation index

**FIGURE 2:**
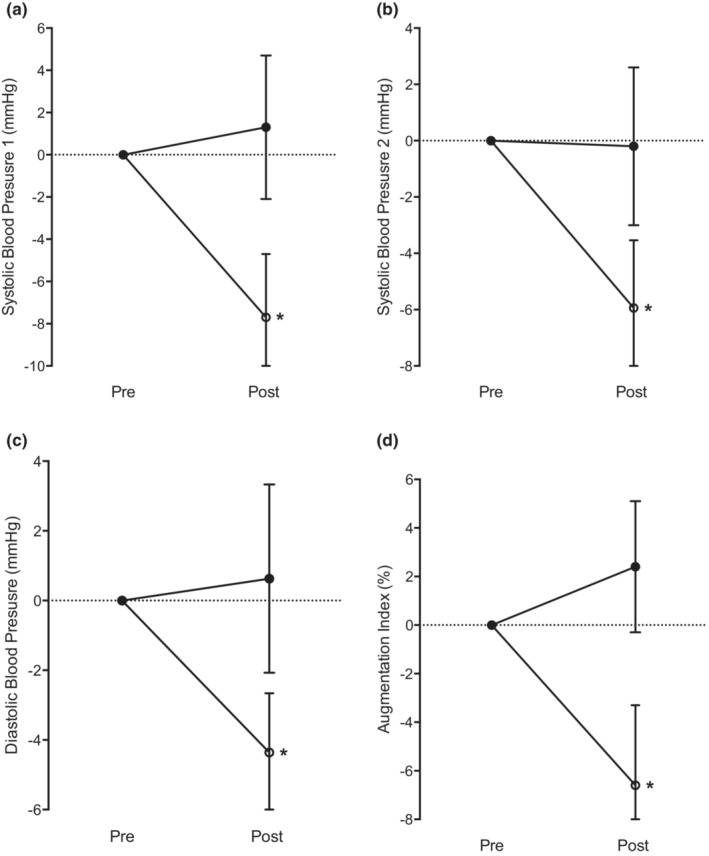
Mean early sBP (a), late sBP (b), dBP (c) and AIx (d) changes following control (closed circles) and IET (open circles) conditions. *Significant (*p* < 0.05) difference in the control and IET change value.

As detailed in Table 1 and Figure 2, sBP 1 (−7.7 ± 12.8 mmHg, *p* = 0.024), sBP 2 (−5.9 ± 9.9 mmHg, *p* = 0.042) and dBP (−4.4 ± 7.2 mmHg, *p* = 0.037) all significantly decreased following IET compared to the control period. There was a significant reduction in AIx following IET compared to the control period (−6.6 ± 14.5%, *p* = 0.02).

**TABLE 1:** Blood pressure and augmentation index pre and post isometric exercise training and control period.

	Control (*n* = 17)		IET (*n* = 17)	
	Mean ± SD		Mean ± SD	
	Pre	Post	Pre	Post
Heart rate (b⋅min^−1^)	63 ± 7.3	62 ± 6.5	64 ± 7.9	62.7 ± 7.9
sBP 1 (mmHg)	113.6 ± 12.8	114.9 ± 12.4	116.1 ± 17.5	108.4 ± 16.4*
sBP 2 (mmHg)	98.7 ± 12.2	98.5 ± 18.3	100.4 ± 18.6	94.5 ± 15.4*
dBP (mmHg)	74.5 ± 13.6	75.1 ± 15.1	78.2 ± 15.1	73.8 ± 12.6*
AIx (%)	39.2 ± 15.7	41.6 ± 15.9	44 ± 15.8	37.4 ± 15.7*

Abbreviations: AI, augmentation index; dBP, diastolic blood pressure; sBP, systolic blood pressure.

* Indicates a statistically significant (*p* < 0.05) difference in the pre to post change value between control and IET intervention group.

Resting clinic sBP (−9.6 ± 4.6 mmHg, *p* < 0.001), dBP (−5.7 ± 5.4 mmHg, *p* = 0.048) and mBP (−7.1 ± 4.6 mmHg, *p* = 0.002) all significantly reduced following IET compared to the control group. Similarly, resting TPR (−140.7 ± 250.0 dynes·cm‐5, *p* = 0.042) and PP (−3.8 ± 4.2, *p* = 0.003) both significantly reduced after IET compared to the control group. Resting SV significantly increased (5.1 ± 3.0 mL, *p* < 0.001) following IET compared to the control group, with no significant changes in resting heart rate or cardiac output.

## 5 DISCUSSION

While the efficacy of IET as an anti‐hypertensive intervention is well‐reported, very little research to date has investigated its potential effects on measures of arterial stiffness. This study demonstrates that a short‐term IET intervention produced statistically significant improvements in the AIx, which is a marker of systemic arterial stiffness, concurrent to significant reductions in TPR and PP (Fantin et al., 2007; Vlachopoulos, Aznaouridis, O'Rourke, et al., 2010). Mechanistically, these improvements, adjacent to a lack of change in CO, indicate vascular adaptations as the primary means for the observed BP reductions.

To date, only few studies have investigated the effects of IET on the AIx. Similar to the present study, Okamoto et al. (2020) included a cohort of unmedicated physically inactive participants and reported significant improvements in AIx following 8 weeks of IET. Conversely, in a group of medicated hypertensives, Farah et al. (2018) found no significant AIx changes after 12 weeks of IET. More recently, Correia et al. (2020) recruited a cohort of medicated patients with peripheral artery disease, and reported no effect on AIx from an 8 week IET intervention. However, the vascular abnormalities and arterial stiffness in patients with peripheral artery disease complicates the possible inferences made from such data. In conjunction with the results of the present work, these studies suggest a potential confounding effect of baseline medication status on the efficacy of IET in improving arterial stiffness. This is likely attributed to the complex overlapping and possibly antagonistic mechanisms driving the observed anti‐hypertensive effects of both IET and cardiovascular pharmacotherapy (Millar et al., 2014). Indeed, a plethora of data has shown significant reductions in the AIx and other parameters of arterial stiffness from varying anti‐hypertensive drug classes (Dudenbostel & Glasser, 2012). Traditional aerobic exercise training has been shown to significantly improve both pulse wave velocity and AIx, with higher intensity exercise demonstrating greater improvement (Ashor et al., 2014). Previous IET research on wider measures of arterial stiffness, such as pulse wave velocity, demonstrate similar findings to that of AIx (Correia et al., 2020; Farah et al., 2018). The consistency of these findings across different methodological approaches to arterial stiffness measures provides support for the application of peripheral AIx as a practical measure. However, adaptations following IET may also be intensity dependent, since Farah et al. (2018) and Correia et al. (2020) found no significant change in pulse wave velocity or AIx following isometric hand grip training, and isometric wall squat training has been shown to be a greater hemodynamic stress in comparison (Swift et al., 2022).

Interestingly, with this study employing a wall squat IET protocol, and given that BP was measured at the index or middle finger and brachial artery, our findings must reflect some degree of systematic change in vascular resistance, rather than localized adaptations to the exercised muscle, as has been previously demonstrated through flow‐mediated dilatation testing following handgrip IET (Correia et al., 2020). While the current evidence surrounding the specifics of vascular adaptation to IET is rather conflicted, the present study supports the notion that IET primarily induces BP reductions via decreases in peripheral vascular resistance, as evidenced through our significant reduction in TPR (Taylor et al., 2019). Specifically, previous work from Taylor et al. (2019) demonstrated these TPR improvements occur concurrent to changes in biomarkers associated with anti‐inflammatory and improved endothelial function, measured through serum interlukin‐6 and asymmetric dimethylarginine. Combined with the findings of the present study, it may be speculated that reductions in BP following short‐term IET are, at least somewhat, modulated via improvements in endothelial function with a potential role of nitric oxide synthase on arterial stiffness and function (Millar et al., 2014; Taylor et al., 2019). In addition, it has previously been suggested that chronic enhancements in anti‐inflammatory regulation may also contribute to reduced arterial stiffness through an improvement in the synthesis and degradation of collagen and elastin, which remain the key scaffolding proteins of arterial structure and stiffness (Zieman et al., 2005). However, in the only other study to investigate serum inflammatory biomarker responses to IET, no changes were found in any parameters in a population of medicated hypertensives (Cahu Rodrigues et al., 2020), lending further support to the previously outlined hypothesis of a medication‐dependent moderator effect. As such, while large‐scale investigations with participant stratification based on medication status are certainly needed, it may be theorized that BP and arterial stiffness reductions following IET could occur in unmedicated participants (ranging from normal to grade 1 hypertensives), through complex changes in chronically regulated systemic structural remodeling, endothelial‐dependent mechanisms, and/or functional adaptations in autonomic vasomotor control. Unfortunately, without larger‐scale direct evidence, the speculative nature of such hypotheses should be acknowledged. While there is reliable data on localized endothelial functional adaptations to IET (Badrov et al., 2016; McGowan et al., 2007), changes in functional vasomotor control are based on indirect measures of autonomic function and structural remodeling adaptations remain speculative due to a lack of long‐term research.

Fundamentally, the AIx reduction seen in the present study is generally greater, or at least similar to that of previous investigations of differing exercises modes. A previous meta‐analysis reported a non‐significant mean reduction of 3% in the AIx from dynamic resistance training (Evans et al., 2018). Separately, a meta‐analysis on the effects of aerobic exercise found significant AIx reductions by 4.3% in healthy adults, and 4.6% in hypertensives (Ashor et al., 2014). Importantly, for every 10% reduction in AIx, there is a 32% decreased risk of an adverse cardiovascular event, and given that we found a 6.6% reduction, this may be associated with a 21% reduced cardiovascular disease risk following 4 weeks of wall squat IET (Vlachopoulos, Aznaouridis, O'Rourke, et al., 2010). However, it should be noted that current AIx prognostic data is largely driven by central measures of stiffness as opposed to the peripheral methodology applied in this work (Vlachopoulos, Aznaouridis, O'Rourke, et al., 2010). While previous research has successfully applied the peripheral AIx, the superior prognostic capacity of central measures are well‐established (Munir et al., 2008). As such, future research is needed to truly understand the translation of peripheral AIx adaptations into clinical outcomes.

### 5.1 Limitations

Interpretation of these findings must be viewed in the context of certain limitations. Firstly, this study was single centre with a limited participant population size including only male participants. Therefore, multi‐centre trials of larger sample sizes with female recruitment are warranted. Additionally, this work was only 4 weeks in duration and thus the longitudinal implications of these findings is limited, especially considering the temporal process of mechanisms such as vascular structural remodeling. It should also be considered that the prognostic utility of upper‐limb exclusive arterial stiffness measures is not entirely clear. The lack of female recruitment also highlights the future need for investigations into the potential sex differences in vascular adaptations following IET. Finally, given the role of aging on arterial stiffness, future research is needed in older adults who are most likely to benefit from such vascular adaptations.

## 6 CONCLUSION

This study demonstrates an improvement in arterial stiffness, measured through the AIx, following a short‐term IET intervention. These findings may have important clinical implications regarding cardiovascular disease risk. Furthermore, these results suggest that reductions in resting BP following IET are induced via favorable vascular adaptations, although the precise mechanisms inducing such adaptations are not yet clear.

## AUTHOR CONTRIBUTIONS

Jamie M. O'Driscoll, Jamie J. Edwards, and Navazh Jalaludeen, conception and design of research; Jamie M. O'Driscoll, Jamie J. Edwards, and Navazh Jalaludeen, performed experiments; Jamie M. O'Driscoll, Jamie J. Edwards, and Arian Beqiri analyzed data; All authors interpreted results of experiments; Jamie M. O'Driscoll and Jamie J. Edwards, prepared figures; Jamie M. O'Driscoll, Jamie J. Edwards, Navazh Jalaludeen, and Arian Beqiri, drafted manuscript; All authors edited and revised manuscript, and approved final version of manuscript.

## FUNDING INFORMATION

None.

## ETHICS STATEMENT

This research study conformed to the Declaration of Helsinki principles and was approved by the local ethics committee (Ref:12/SAS/122).
